# The Opioid Epidemic: A Review of the Contributing Factors, Negative Consequences, and Best Practices

**DOI:** 10.7759/cureus.41621

**Published:** 2023-07-10

**Authors:** Dallin Judd, Connor R King, Curtis Galke

**Affiliations:** 1 Medicine, Texas College of Osteopathic Medicine, University of North Texas Health Science Center, Fort Worth, USA; 2 Marriott School of Business, Brigham Young University, Provo, USA; 3 Family Medicine and OMM, Texas College of Osteopathic Medicine, University of North Texas Health Science Center, Fort Worth, USA

**Keywords:** substance abuse, prescription drug monitoring program, opioid-related death, drug addiction, opioids use

## Abstract

The opioid epidemic is a significant public health crisis that has caused extensive harm and devastation in the United States. This literature review aimed to identify the contributing factors and negative consequences of the epidemic, as well as best practices for healthcare providers in managing the epidemic. Overprescribing opiates and opioids, lack of education and opportunity, and being unmarried or divorced were some of the identified contributing factors to dependence on opioids. The epidemic's negative consequences are substantial, leading to increased access to opioids for vulnerable populations, which consequently cause accidental death among men and the degradation of rural community health services. As part of the literature review, we also analyzed the best practices for healthcare providers, including implementing prescription drug monitoring programs (PDMPs). However, we found that while PDMPs resulted in a decrease in opioid overprescription and an increase in provider confidence when prescribing medication, the evidence for their effectiveness in improving rural community health services or reducing opioid overdoses and opioid-related deaths was inconclusive. Our review highlights that the greatest challenge to overcome is a lack of legal mandates and proper education for healthcare providers on best practices for addressing the epidemic. To regulate and control opioids effectively, tracking and standardizing prescription models by federal agencies and medical institutions is necessary but not enough. Legal action is vital for the successful containment of the opioid crisis.

## Introduction and background

Drug overdose is the leading cause of accidental death in the United States, with accidental deaths being the fourth highest cause of death in the country [1]. The majority of overdose deaths involve opioids [2]. Although the opioid epidemic has claimed many lives of people of all ages over the years, those in the 35-44-year age group experience the greatest number of opioid-related deaths, with 71% of all opioid-related deaths occurring between the ages of 25-54 years [3]. Of those deaths, nearly seven out of 10 were male. These men were typically less educated, divorced or unmarried, unemployed, and lived in rural regions of the United States [4]. 

Since opioids became broadly available in the 1990s and the tracking of associated deaths began in 1999, rates of opioid-related deaths have tripled. Though opioid-related deaths have regularly increased since they were first tracked in 1999, 2020 saw a 41% increase in opioid-related deaths, possibly related to the coronavirus disease 2019 (COVID-19) pandemic [5]. Despite the general increase in these deaths in both genders, men remained disproportionately affected.

Despite the rise of opioid-related deaths worldwide, the United States has the highest rate of opioid consumption per capita; the United States also experiences the highest number of opioid-related deaths. This review will analyze various factors associated with the epidemic in rural, non-rural, and urban areas of the United States. However, our focus will be on the rural regions of the nation as they see a greater frequency of opioid-related deaths and abuse [6].

A further breakdown of the primary victims of the opioid epidemic reveals that Caucasians, Native Americans, and Alaska Natives are the most at-risk populations [6]. Nearly 60% of opioid-related deaths occur among individuals who failed to attain or only attained a high-school diploma or GED [4]. While African-American and Hispanic communities are similarly experiencing an increase in opioid-related deaths, the highest rates of opioid-related deaths are reported from communities in the Northeast, Mountain states, Midwest, and Appalachian regions of the United States, which are predominantly white demographically [7]. The major risk factors for opioid abuse include a previous history of substance abuse, untreated psychological disorders, younger age, and a social or family environment that encourages misuse [8].

## Review

This review aims to comprehensively analyze the opioid epidemic in the United States by identifying the contributing factors and negative consequences as well as best practices for healthcare providers in managing the crisis. To ensure a thorough review process, two researchers independently conducted literature reviews on the subject matter using databases such as PubMed and Google Scholar. The search criteria included a range of relevant keywords such as "contributing factors of the opioid epidemic", "negative consequences of the opioid epidemic", and "best practices for healthcare providers in managing the opioid epidemic". The researchers combined their findings and reconciled any discrepancies after their respective reviews. Studies that did not meet the inclusion criteria were excluded, and the most relevant papers were selected for the review. The collected papers provided an insightful and detailed analysis of the opioid epidemic; this analysis offers valuable insights into the nature and scope of the crisis.

Contributing factors

Overprescription of Opiates and Opioids

Pharmaceutical companies that produce the drugs, the pharmacies that distribute prescription drugs, and the doctors who prescribe them all share in the blame for the opioid crisis. The overprescription of opioids has significantly contributed to the opioid crisis [9,10,11]. From 1999 to 2008 alone, there was a fourfold increase in prescription opioid sales, associated with a fourfold increase in deaths attributed to prescription opioids [5]. This increase occurred partly because of a hospital culture shift, particularly with the changes to the "vital signs" physicians used to diagnose a patient's condition. In the 1990s, external authorities such as the American Pain Society, the Veterans Health Administration, and The Joint Commission proposed that physicians use a patient's self-reported pain level as a "fifth vital sign" [5]. At the same time, hospital administrators sought to increase patient satisfaction and adopted these guidelines; in 2006, they would again change the medical culture by implementing patient surveys that gave feedback on how they felt their pain was being managed. The results of these surveys were publicized and allowed comparisons between hospitals, which could influence hospital funding. In addition, these external forces encouraged the liberal use of opioids in treating patients' pain, where, despite the risks involved, a physician may be encouraged to issue a generous amount of a particular opiate-based painkiller as long as it means that patient satisfaction may increase. Perhaps, most interesting of all is the effect that the regulation of opioids has had on physicians' willingness to overprescribe opioids, especially in rural areas where patients may travel long distances for treatment or lack alternative pain management solutions. Since opioids can only be supplied with a prescription note, frequent patient consultations can take much of a physician's time. Some patients may only manage to have one long-distance trip to the hospital or care facility; thus, it becomes more convenient for a physician to prescribe large amounts of opioids to save both parties time [12].

Lack of Education and Opportunity

Other than intrinsic traits such as sex and race, the most outstanding issue plaguing opioid overdose victims is the lack of education. According to a study published in 2020, people whose highest educational attainment was a high school diploma or GED accounted for 35.4% of all opioid-related deaths. Those who failed to receive a GED or finish high school accounted for 23.7% of opioid overdose deaths [4]. With the evolution of the United States job market and the growing shortage of stable, sustainable jobs for those who lack college education or vocational training, many individuals without education are typically left with few job opportunities that allow them to advance financially and professionally. The unskilled labor market has traditionally relied heavily on the manufacturing sector to provide jobs that guarantee good pay and benefits, attributes associated with such jobs' unionized nature. However, the "outbreak" of the opioid epidemic in the late 1990s coincided with a decline in manufacturing jobs. According to the Bureau of Labor and Statistics, manufacturing jobs have decreased by 35% from 17.2 million to only 12.5 million in 2018 [13]. This lack of relatively well-paying, low-barrier-to-entry opportunities has been increasingly replaced by service-industry jobs that do not provide many of the benefits and pay many of these unskilled laborers have relied on. These unskilled laborers, therefore, become a more vulnerable population, statistically more likely to engage in substance abuse that can escalate into overdose.

Being Unmarried or Divorced

The rates for opioid-related deaths are disproportionately large among those who are unmarried or divorced. The same study mentioned previously has found that people who had never married accounted for 42.9% of all opioid overdose deaths, while divorced individuals accounted for 21.3% of these deaths [4]. Loneliness is often termed the "silent killer" and has been shown to have negative mental health consequences, often precipitating depression or suicidal ideation. Buprenorphine is an opioid-based medication primarily used for treating opioid use disorder (OUD), acute pain, and chronic pain. Recently, it has also been increasingly prescribed for the treatment of depression. According to the National Institutes of Health (NIH), drug misuse has decreased in the past few years, though many instances of drug abuse have led to overdose. This is an ongoing area of study related to the opioid crisis, and it will take more research to uncover precisely why this link is so strong [14,15,16].

Negative consequences

Leading Cause of Accidental Death Among Men

Since the tracking of opioid-related deaths began in the late 90s, the proportion of deaths from opioid overdose has increased from 0.4% of all deaths in the United States to 1.5% in 2016, representing a 292% increase [17]. The leading causes of death in the United States in 2020 - heart disease, cancer, and COVID-19 - were seen fairly equally in both genders; however, the fourth leading cause of death, accidental death, has not seen such parity across the sexes. As discussed previously, accidental deaths in the United States are primarily opioid-related, of which men account for nearly 70% [4]. Although the rates of opioid-related deaths have increased at a faster rate among women in the United States than men since 1999, at a rate of 1,608% for women and 1,076% for men [3], adjusting for increases in rates of death among homeless white women, men still remain the most at-risk population overall [18]. Those who are unmarried or widowed are one of the most vulnerable populations among men; men who, when addicted to opioids and who suffer death by overdose, are termed to have died a "death of despair," a metric indicative of the United States' growing loneliness epidemic [19]. Clearly, the United States is facing an epidemic of opioid-related deaths [20,21,22].

Increased Access to Opioids for Vulnerable Populations

Due to the fourfold increase in opioid sales from 1999 to 2008 and the sharp surge in prescription opioid sales since 2008, many Americans are inundated by a small number of opiate-based drugs for nearly all medical conditions [12]. Among adults in the United States, an estimated one in three are currently using or have used some form of a prescription opioid, with another estimated 4.7% abusing them [23]. After the introduction of synthetic opioids as an effective means of pain management in the late 90s, the most common opiate-based painkillers in the United States have been morphine, Vicodin, OxyContin, methadone, and fentanyl. While the United States makes up 4.4% of the world's population, it consumes over 80% of the world's opioids [24]. According to a study published in the British Journal of Urology (BJU), in 2015 alone, there were enough prescription opioids dispensed in the United States to medicate every adult with 5 mg of Vicodin every four hours for three weeks, a dosage powerful enough to remain in the body for up to 24 hours [5]. The reasons for the popularity of these drugs have previously been expounded upon in earlier sections of this brief, yet what is perhaps most interesting about the panacean treatment of these drugs is how many go unused. The same BJU study found that 71% of all opioids prescribed to patients who had undergone thoracic, gynecological, upper extremity, urological, dental, and cesarean-section procedures were never used. Indeed, after analyzing the prescriptions given to patients, it was found that the prescribed number of pills exceeded the number of used pills by about 200% [5]. With just over 50% of all people with OUD obtaining their supply of drugs from relatives with a prescription, overprescribing prescription opioids continue to fuel the epidemic across the nation [25].

Degradation of Rural Community Health Services

Rural communities have been the epicenter of the opioid epidemic for decades, through the various waves of the opioid epidemic that have affected the United States across various demographics [26]. The strain of treating OUD puts rural hospitals in a precarious position due to the lack of experienced professionals who can effectively treat the condition as well as the expensive nature of the materials required for treatment. Aside from not always being equipped to treat OUD, rural hospitals are also plagued by a lack of reimbursement from their government benefactors, which prevents them from maintaining staff and providing effective care [27,28]. With just over 30% of all Medicaid patients in the United States living in rural areas and being serviced by rural hospitals, the treatment provided to these individuals is reimbursed for less than the cost of treatment by the federal government [29]. Three in 10 individuals with OUD are covered by Medicaid, and one in five are uninsured, meaning over half of all individuals presenting with OUD will require care that brings little to no reimbursement to the hospitals [30]. In 2020 alone, rural hospitals incurred $7 billion in Medicaid and Medicare underpayments and $4.6 billion in uncompensated care [31]. While OUD treatments are not wholly responsible for the pressures these rural hospitals face, the increased care costs per OUD patient placed upon rural hospitals can be devastating. With one in 12 jobs and nearly $220 billion of economic activity in rural communities revolving around hospitals and other healthcare facilities, the consequences of hospital underfunding and potential closure can significantly affect local economies [32].

Best practices

Practice - Altering Physician Prescription Methodology

In order to curb the effects of opioid abuse at the source, efforts have been made recently to not only reduce the amount of opioids prescribed for postoperative recovery but also to track and standardize the prescription of opioids. Greater visibility has been achieved in disseminating Schedule 2-5 controlled substances through implementing prescription drug monitoring programs (PDMPs), which track when and where a prescription is filled and by whom [32]. Since 2001, when only 16 states utilized some form of a PDMP, 49 states have now instituted a PDMP to assist in tracking the controlled substances being prescribed by providers [33]. With the data collected from such programs, researchers have created recommended prescription values that physicians can use when determining how many opioids to prescribe to a patient. Along with tracking prescription opioids and standardizing their use, there have been greater efforts to encourage more strategic perioperative and postoperative care by physicians. Such changes include the decreased prescription of opioids during surgical procedures and utilizing non-narcotic analgesics instead of opioids whenever possible [5]. A study published in the American Journal of Drug and Alcohol Abuse revealed that opioid-addiction patients who were prescribed buprenorphine via telemedicine exhibited superior retention rates. This finding underscores the potential of telemedicine in enhancing access to effective treatment for OUD [34].

Impact

Based on the data gathered from these practices, it has been observed that on a localized scale, PDMPs and standardized prescription models have resulted in a decrease in opioid overprescription as well as an increase in the confidence of providers when prescribing medications. For example, according to a study in the Annals of Surgery, implementing department-wide standardized opioid prescription models led to a 53% reduction in opioid prescribing compared to pre-education levels [35]. Among ophthalmic surgeons and trainees with the Mayo Clinic in Minnesota, there has been a reported decrease in patients who were prescribed opioids - from 4.4% to 3.0% - after instituting a standardized model for issuing prescriptions [36]. In addition, a study among emergency department physicians in Wisconsin found that 97% reported that the implementation of PDMPs has been helpful in identifying patients who had a pattern of drug abuse. As a result, 90% of these physicians prescribed fewer opioids [32]. Similarly, of a group of Maryland physicians participating in a study on the effectiveness of PDMPs, 70% found that they were more comfortable when prescribing opioids and also prescribed fewer due to these programs, and 74% cited them as being important to their decision-making when issuing prescriptions [32]. Though these and other examples demonstrate the efficacy of PDMPs and standardized prescription models in limiting the overprescription of opioids and the access of opioids to vulnerable populations on a local scale, there is little evidence to suggest that these solutions are working equally well across the nation or that they are reducing opioid overdoses and opioid-related deaths [37,38,39]. Furthermore, there is little to no evidence to suggest that these practices improve rural community health services, though some anecdotal examples may exist [40].

Gaps

Despite the efforts of many researchers, physicians, and medical organizations to combat the opioid epidemic through the practices listed previously, perhaps the greatest challenges to overcome are the lack of legal mandates and the failure to properly educate providers on the practices themselves [41,42]. In the study involving Wisconsin emergency department physicians cited above, 36% reported that they did not use the PDMP, citing their limited knowledge of the registration process or lack of a Drug Enforcement Administration number; another 20% who did use the PDMP stated they had issues accessing the data the system collected [32]. A study on the PDMPs of 22 states found no correlation between patients' PDMP status and their admission for heroin or prescription opioid treatment [41]. The researchers concluded that although PDMPs may assist physicians and governmental entities in tracking Schedule 2-5 substances, there was little to no evidence to suggest that they were effective in reducing opioid-related deaths or OUD [33]. The standardization models for prescription opioids have likewise been challenging to implement successfully, even though groups of physicians and medical organizations are pushing for more responsible postoperative care [43,44]. These initiatives often include the utilization of non-narcotic analgesics or changes to the base-30 prescription model that most providers currently use. These changes would necessitate disrupting current pharmaceutical distribution methods [45]. Transitioning from a system of filling prescriptions that are either multiples of 30 or 10 to one involving the numbers recommended by standardization models, such as 7, 17, 19, or 23, would take time to implement successfully. Since this change is not mandated by law and is also not seen as beneficial to pharmacies filling prescriptions, pharmacies have continued to pressure physicians to utilize the base-30 system, or at least multiples of 10, when issuing prescriptions [45].

## Conclusions

The United States has over-relied on prescription opioids and other opiate-based medications for decades, leading to a sharp increase in opioid overdose and opioid-related deaths in the past 25 years. Although the opioid epidemic has impacted all demographics, men have disproportionately been affected, with opioid overdose now being the leading cause of accidental death among men. Additionally, with so many medical resources being used to treat OUD, rural areas will continue to suffer due to a lack of proper government reimbursement for most OUD treatments provided to those primarily insured through Medicare/Medicaid. More extensive tracking of opioids by the federal government and medical institutions and the implementation of standardized opioid prescription models will allow for more regulation and control of how opioids are prescribed. Despite the notable decline in opioid prescriptions, there is no substantial evidence to suggest a corresponding decrease in OUD or overdose deaths. While curtailing prescriptions is a crucial aspect of the strategy against the opioid crisis, further research is needed to fully understand its overall impact on the prevalence of OUD.
